# 

**Published:** 1803-12-01

**Authors:** 


### TO CORRESPONDENTS.

Communications are received from Dr. Reid, Mr. G. N. Hill, Mr. Barlow, Mr. Marson, Mr. Adye, and Mr. Roche.

---

END OF VOL. X.

### INDEX.

ABDOMEN, cafe of a wound of the 250
Aberdeen, account of the influenza at 232
Abergavenny, epidemic at 228
Abernethy's, Mr. lectures announced 286
Aberyftwyth, epidemic at 294
Abfcefs of the liver, cafe of 1
————————, Dr. Robinfon on 489
Abforption, on aqueous 570
Acid, on preparing muriatic æther with fimple 94
———, on the exiftence of cobaltic 94
Ackermann's, Dr. Galvanic experiments 576
Æther, method of obtaining muriatic 94
Africa, on the remittent fever of the coaft of 85
Ague, on that of hot climates 85
Agues, on the ufe of white vitriol in 496
Albugo, defcription and cure of 562
Alderfon, Dr. on the influenza 125
Aldis's, Mr. cafe of fiffure of the cranium 244
Alkalies, on the antifeptic qualities of 475
Allen's, Mr. lectures announced 285
Almonds, pruffic acid in the infufion of bitter 95
Amaurofis, on the application of Galvanifm in cafes of 330
Amblyopia cured by Galvanifm 58
America, quantity of fpirits diftilled in 476
Analogy between the motion of the blood in the arteries, and of water in troughs and conduits 285
Angina pectoris, cafe of 63
Animal flefh, employment of pot-afh with 475
Animalcula, obfervations on 139
Animal matter, on the action of heat or cauftic on *(note)* 26
Animals, caufe of motion and fenfation in 253
————, on contraction in the mufcles of 53
Antyllus on Bronchotomy 7
Aqua laurocerafi, pruffic acid in the 95
Aretæus on bronchotomy 7
Arnemann's, Prof. inftruments for extracting the cataract *(with an engraving)* 65
Arfenic, on its effects on the human body 575
Artery, on the incontraction of an 2
Arteries, on the difficulty of compreffing the great 156
————, on the motion of the blood in the 284
Artificial Cheltenham water, defcription of 574
Afcites, fuccefsful operation in 498
Afthma, on the lichen iflandicus in 379
Atkinfon, Mr. on metallic cafts of the ear 359
Auban, Dr. on the cow-pock, as a prefervative againft the plague 474
Auditory organs, method of applying Galvanifm in affections of the 61
Aurelianus on bronchotomy 7
Autumnal fever, hiftory of the 365
Axilla, on compreffing the greater arteries at the 156

Babington's, Dr. lectures announced 285
Baddeley, Mr. on the influenza 214
Badham's, Dr. lectures announced 384
Baines, Mr. on the influenza 396
Barbadoes, account of the vaccine inoculation at 424
Barbary, obfervations on the coaft of 381
Bardfley, Dr. on the influenza 210
Bark, on the ufes of, in fevers 12, 18
Barlow, Mr. on the influenza 209
Barnfide, Dr. on the influenza 528
Barthe, M. his cafe of a wound in the abdomen 250
Bartley, Mr. on the influenza 308
Baffe, Mr. on muriatic æther 94
Bath waters, review of a treatife on the 183
Batty's, Dr. lectures announced 286
Bayfillance's, Dr. cafe of tinea 542
Beaumont, Dr. on the influenza 202
Beddoes's, Dr. communications on the influenza 73, 97, 193, 289, 385, 517
————, Dr. on a medical inftitution for the poor 571
Bell, Mr. on the influenza 517
Bell's Principles of Surgery, remarks on 154
———, Mr. C. lectures announced 576
Bideford, account of the influenza at 104
Black, Dr. on the influenza 295
Blackburne, Dr. on fcarlet fever, reviewed 457
Bladders, on the cure of difeafed 82
Blair's, Mr. cafe of an extraneous fubftance lodged within the vagina 491
————, lectures announced 287
Blake, Dr. on the influenza 116
Bleeding, its ufe in fever 14
Blifters, on their ufe in puerperal fever 17
Blood, on an iffue of the arterial 4
———, on the motion of the 284
Blood veffels, on the compreffion of the large 154
Blount, Dr. on the influenza 126
Blue difeafe, account of the 575

Boarding fchools, advice to the heads of 458

Bones, on a caries of the 4

Books, critical analysis of medical—The Natural Hiftory of the Human Teeth, by Jofeph Fox, 77. Account of the Difcovery of the Power of Mineral Acid Vapours to deftroy Contagion, by John Johnftone, M. D. 78. On the Influenza as it prevailed at Briftol and its Vicinity, by John Nott, M. D. 80. Cafes of the fuccefsful Practice of Veficæ Lotura for the Cure of difeafed Bladders, by Jeffe Foot, Efq. 82. Medical Directions for the Ufe of Navigators and Settlers in Hot Climates, by Thomas M. Winterbottom, M. D. 83. Illuftrations of fome of the Injuries to which the Lower Limbs are expofed, by C. B. Trye, 88. An Enquiry into the Efficacy of Oxygen in the Cure of Syphilis, by C. Platt, 90. Ufeful Hints to thofe who are afflicted with Ruptures, and on the Empirical Practices of the prefent Day, 92. The Edinburgh Practice of Phyfic, Surgery, and Midwifery, a new edition, in 5 vol. 180, 277. A Second Treatife on the Bath Waters, by George Smith Gibbes, M. D. 183. The Report on the Cow-Pock Inoculation, from the Practice at the Vaccine-Pock Inftitution during the years 1800, 1801, and 1802, 187. Obfervations on Diarrhœa and Dyfentery, as thofe Difeafes appeared in the Britifh Army during the Campaign in Egypt, in 1801, by Henry Dewar, 279. A concife Hiftory of the Autumnal Fever which prevailed in the Borough of Wilmington, in North America, in the Year 1802, by Dr. John Vaughan, 365. Practical Obfervations on Surgery, illuftrated with Cafes, by William Hey, 369, 466. Experiments and Obfervations on the Cortex Salicis Latifoliæ, or Broad-Leafed Willow Bark, by G. Wilkinfon, 374. Practical Obfervations on the Management of Ruptures, by W. H. Timbrel, Efq. to which are prefixed, two recommendatory Letters, by William Blair, A M. 375. Facts and Obfervations concerning the Prevention and Cure of the Scarlet Fever, with fome Remarks on the Origin of acute Contagions in general, by W. Blackburne, M. D. 457 The Charlefton Medical Regifter, for the Year 1802, by David Ramfay, M. D. 468. A Treatife on the Cow-Pox, containing the Hiftory of Vaccine Inoculation, and an Account of the various Publications which have appeared on that Subject, by John Ring, 473. The Elements of Phyfiology, by M. Richerand, 567. Rules of a Medical Inftitution for the Poor at Briftol, by T. Beddoes, M. D. 571

Books, new medical announced.—Mr. Henry's third Edition of his Epitome of Chemiftry, 96. Dr. Percival's Medical Ethics, *ib.* Mr. Dewar's Obfervations on Diarrhœa and Dyfentery, *ib.* Dr. Blackburne's Obfervations on the Prevention and Cure of Scarlet Fever, 384. Mr. Johnfton's Hiftory of Animal Chemiftry, 480. Mr. Skrimfhire's Popular Chemical Effays, *ib.*

Bradford, account of the epidemic at 387

Bradley's, Dr. lectures announced 286

Breaft, cafe of abfcefs in the 414

———, cafe of cancer of the 448

Bree, Dr. on the influenza 125

Brewer, Mr. on the influenza 522

Bridgewater, epidemic at 228

Briftol, on the influenza in 80

Bronchotomy, on the operation of 6

Broufonet, Dr. on fcarifying and cupping 425

Brown, Mr. on the influenza 118

Brunonianifm, obfervations on 181

Buchholz, M. on the analyfis of opium 42

——————— on cobaltic acid 94

Burfæ Mucofæ, difeafe of one of the 69

Bufh's, Mr. cafe of dropfy 498

Callanan, Dr. on the influenza 523

Cambridge, Mr. on the influenza 203

Cambridgefhire, account of the epidemic in 205

Cancer, cafe of 448

Carendeffez, M. de, on ftony concretions 328

Carey, Mr. on the influenza 529

Caries of the joints, on amputating the extremities of the bones in cafes of 557

Carlifle's, Mr. lectures announced 384

Carmarthen, influenza at 303

Carolina bank, extraordinary attempt to rob 472

Carpue's, Mr. Galvanic experiments 383

———————, Lectures announced 287

Cataract, on the cure of 564

————, cafe of early 10

————, account of inftruments for extracting 65

————, account of a couching needle for 371

Catarrhal peripneumony, on cupping in 425

Catheter, method of fecuring the elaftic 174

Cats, diforder among 217

Cerufſa acetata, its ufe in hæmorrhage 236

Cevallos, M. on the goat pock 341

Chamberlaine, Mr. on the medicine act, 165, 258, 347

Chancre, on the cure of 499

Charcoal, remarks on experiments on 273

Charlefton Medical Regifter, review of the 468

Cheltenham waters, on artificial 574

Chemiftry, lute for the operations of 192

Chemofis, on the cure of 562
Chevalier's, Mr. lectures announced 287
Church-yard infection, remarks on 177
Cinder, remarks on finery 278
Clark, Dr. on the influenza 202
Clarke's, Dr. lectures announced 286
Climates, on the fevers of hot 36
Cline's, Mr. lectures announced 285
Clofe-ftools, obfervations on 176
Cobalt, method of obtaining metallic 95
Cobaltic acid, on the exiftence of 94
Cock, Mr. on the influenza 395
Cold water, its ufe in convulfions 410
———, its ufe in opifthotonos 492
Compound fracture, cafe of 316
Concretions, on ftony 327
Contagion, on the fuppreffion of 460
———, on the difcovery of the power of mineral acid vapours to deftroy 78
Contagious difeafes, the re-appearance of 177
Convulfions, on the ufe of cold water in 410
Cook, Mr. on the influenza 197
Cooke's, Mr. cafe of incontinence of urine 423
Cooper, Mr. A. lectures announced 285
Cornea, on the cure of ulcers of the 563
Correfpondents, addreffes to, 96, 192, 288, 384, 480, 576
Cortex falicis latifoliæ, experiments on the 374
Cow-pock, on the origin of the 93
———, inoculation, report on the 187
———, a fecurity againft the plague 474
———, fheep inoculated with the 192
———, account of in Barbadoes 424
———, Mr. Wefton on the 544
Cow-pox, review of Mr. Ring's Treatife on the 473
Cranium, cafe of fiffure of the 244
———, on fractures of the 370
Creafer, Mr. on the influenza 114
Crediton, epidemic at 311
Creve, M. on the metallic ftimulus 54
Crumpe, Dr. on inteftinal vermes 550
Cruikfhank's, Mr. expetiments; Dr. Prieftley's remarks on 273
Cuming, Mr. on the influenza 312
———, on the ufe of white vitriol in agues 496
Cupping, on the ufe of 425
Currie, Dr. on the influenza 213
Curry's, Dr. lectures announced 285
Cuftance, Mr. on the influenza 200
Cutaneous difeafes, efficacy of muriatic acid in 576

Davies, Mr. on the influenza 105
———, Dr. on ditto 303
Davis's, Dr. obfervations on the Barbary Coaft 381
Deacon, Dr. letters from Dr. Mead to 48
Deafnefs, on the benefit of Galvanifm in 61, 332
De Carro, Dr. on vaccination 242
———, letter to Dr. Jenner from 274
Deiman, Dr. on furoxygenated muriatic acid 576
Delaferronnays. Baron, on the Iceland Lichen 379
Delunel, M. on inodorous plants 95
Dennet, Mr. on inoculation 321
Dennison's, Dr. lectures announced 286
Dewar, Mr. on the influenza 119
———, his obfervations on diarrhœa and dyfentery, reviewed 279
Diaphoretics, on their ufe in puerperal fever 16
Diarrhœa, obfervations on 276
———, the radix lopez recommended in 477
Digeftion, obfervations on 318
Digitalis, on its fuccefs in phthifis 551
——— its inefficacy in ditto 554
Difeafes, cafes of general ones cured by the extraction of decayed and difeafed teeth 431
Difeafe of one of the burfæ mucofæ 69
Difeafes, on contagious 177
Difeafes in an eaftern diftrict of London, from May 20 to June 20, 1803 76
———, from June 20 to July 20 180
———, from July 20 to Auguft 20 276
———, from Auguft 20 to Sept. 20 378
———, from Sept. 20 to Oct. 20 456
———, from Oct. 20 to Nov. 20 566
Diflocations, remarks on 89
———, cafe of 467
Dixon, Dr. on the influenza 110
Dogs, experiments on 22
———, affected by the influenza 217
Dolor faciei, cafe of 420
Doyle, Dr. on the influenza 193
Drogheda, epidemic at 527
Dropfy, cafe of 498
Drunkennefs, remarks on 382
Dugard, Mr. on the influenza 215
Dumas, Mr. on the caufe of hunger and thirft 20
———, his cafe of intermittent fever 427, 541
Duncan, Dr. on the influenza 405
Dunn's, Mr. cafe of phthifis pulmonalis 551
Dunning, Mr. on the influenza 129
Dyfentery, obfervations on 280

Ear, on metallic cafts of the 171, 357, 359
———, on applying Galvanifm to the 62
Earneft, Mr. on the influenza 387
———, cafe of phthifis pulmonalis 554
Eaft Bourn, account of the epidemic at 127
Edgeworth, Mr. on the influenza 301
Edinburgh, account of the epidemic in 405

Edinburgh claffes 576
Edinburgh practice of phyfic, furgery, and midwifery, reviewed 186, 277
Edlin, Mr. on the petechiæ fine febre 444
Egypt, on the difeafes of 279
——, remarks on the climate of 465
Electricity, parallel between Galvanifm and 331
Elements of phyfiology, review of the 567
Emetics, their ufe in fevers 15, 38
Emhoff, Mr. on Galvanifm 59
Enemas, their ufe in fevers 39
Epidemics, on the production of—fee INFLUENZA 140
Epilepfy, hiftory of a cafe of 51
————, cured by Galvanifm 332
Evaporation, account of an inftrument for facilitating 479
Excitability, on the caufes of 25, 252
Experiments on the caufe of hunger and thirft 22
——————, relative to the analyfis of opium 42
——————, on the irritated mufcular and nervous fibre 55
——————, on cobaltic acid 94
——————, on the application of Galvanifm for medical purpofes 330
——————, with oxydated difks of the Galvanic pile 63
——————, remarks on Mr. Cruikfhank's 273
—————— and obfervations on the cortex falicis latifoliæ 374
——————, Dr. Ackermann's 576
——————, Mr. Carpue's Galvanic 383
Extraction of decayed teeth, on the 431
Extraneous fubftance lodged in the vagina, cafe of 491
—————— bodies, account of an inftrument for extracting them from the œfophagus 479
Eyes, obfervations on feveral difeafes of the 561
Fabroni, Mr. on Galvanifm 54
Faciei, cafe of dolor 420
Facts and Obfervations on the prevention and cure of fcarlet fever 457
Fahrenheit, mode of converting the degrees on Reaumur's thermometer to thofe on, *note* 381
Febris hydrophobica, cafe of 427
Femur, cafe of the luxation of the 494
——, on the diflocation of the 88
Fergufon, D. on the cure of chancre 499
Fever, on the nature and cure of the puerperal 11
Fevers, on the ufe of gelatine in intermittent 323
——, of hot climates, obfervations on the 36
——, on the cure of 85
——, on the yellow 87
——, hiftory of the yellow 266
Fevers, hiftory of the autumnal 365
——, cafe of variety of intermittent 427, 541
——, obfervations on the nettle-rafh 481
——, on the prevention of fcarlet 457
Field, Mr. on the influenza 223
Fieldhoufe, Mr. on the influenza 399
Finfbury difpenfary, report of furgical cafes in the 74, 178, 275, 376
Flannel, on the ufe of 84, 281
Foot, Mr. on difeafes of the bladder, reviewed 82
Foffile fifh, difcovery of a beautiful 480
Fothergill, Mr. on the influenza 393
Fox's, Mr. natural hiftory of the teeth reviewed 77
————, remarks on, by Mr. Moor 360
——, lectures announced 480
Fowler, Dr. on the influenza 389
Fracture, cafe of compound 316
Frampton's, Mr. lectures announced 286
Frazer, Dr. on the influenza 204
Friend's hiftory of phyfic, a quotation from 6
Frogs, Galvanic experiments on 57
Fungus hæmatodes, obfervations on the 466
Furniture of a fick room, remarks on 175

Gaillard, Mr. on preferving vaccine virus 41
Galvanic difcovery, hiftorical ftatement of 53, 330
———— pile, experiments with oxydated difks of 63
———— experiments 383
Galvanifm, M. de la Melhere on 26
————, parallel between electricity and 331
————, on the medical benefits of 330
Gangrenes, cafes of 275
Gelatine, on its ufe in intermitting fevers 323
Gibbs, Dr. on the Bath waters 183
Gibney, Dr. on the influenza 527
Gidely, Mr. on the influenza 294
Gilbert, Mr. on gelatine in intermittent fevers 323
Giraud's, Mr. cafe of Monftrofity *(with an engraving)* 171
Glaftonbury, account of the influenza at 226
Goat-pock inoculation, papers on 339
Gonorrhœa, bad effects of oxygenated muriat of mercury in 283
Goodwin, Mr. on the influenza 195
Gottingen, queftion propofed by the academy of 383
Gout, fuccefs of cold affufion in 254
——, on reduced temperature in 357
——, on the frigorific procefs in 377
Grant, Dr. on the influenza 305
Gregory, Dr. account of the death of 530
Griffith, Mr. on the influenza 101
Griffiths, Mr. on ditto 497

Grimm, Profeſſor, on Galvaniſm 58

Groin, on compreſſing the arteries at the 156

Guttfeldt, Dr. on the Yellow Fever 383

Hadley, Mr. on the influenza 212

Haighton's, Dr. lectures announced 285

Haines, Mr. on preſerving vaccine virus 40

Hall, Mr. on the influenza 220

Halliday, Dr. on the influenza 301

Hanſard, Mr. on the influenza 97

Harneſs, Mr. on the influenza 291

Harries, Dr. on the influenza 112

Harriſon's, Mr. caſe of dolor faciei 420

Head-ach, effects of Galvaniſm in the 332

Headington's Mr. lectures announced 286

Heat, on the action of 25

Heberden, Dr. on the Bath Waters 183

Heinze, Dr. inoculates ſheep with the cow-pock 192

Hellwag, Dr. on the medical application of Galvaniſm 333

Henry's, Mr. Epitome of Chemiſtry noticed 96

Hereford, epidemic at 107

Hernia, on an incarcerated 4

———, obſervations on a caſe of 248

———, on the treatment of ſtrangulated 373

——— congenita, caſe of 33

Hey, Mr. on the influenza 194

———, his obſervations on ſurgery reviewed 369, 466

Heydeck, Mr. on the goat pock 339

Hickins's, Mr. caſe of mania 50

Hill, Mr. on the influenza 195

———, G. on opium 532

Hindoos, their eagerneſs to receive the cow-pock 93

Hird, Dr. on the influenza 518

Hiſtorical ſtatement of the Galvanic diſcovery 53, 330

Hiſtory of the yellow fever 266

——— of the autumnal fever 365

Hobbes, Dr. on the influenza 289

Hodge, Mr. on the influenza 197

Holder, Dr. on vaccination 424

Hooſt, a diſorder among cats 217

Hot climates, on the fevers of 36

Hughes, Dr. on the influenza 107

Hugo, Mr. on the influenza 311

Humboldt, M. on Galvaniſm 55

Humerus, on the reduction of the 90

Hunger, on the cauſes of 20

Hunter, Mr. on the influenza 233

———, J. on preparing caſts of the ear *(note)* 359

Huxham, Dr. on an epidemic 135

Hydatids, abſceſs of the liver, with a formation of 489

Hydrocephalus, caſes of 74

——— ——— internus, caſe of 150

Hydrophobic fever, caſe of an intermitten 427

Illuſtrations of the injuries to which the lower limbs are expoſed 88

Incarcerated hernia during pregnancy, caſe of 4

Income tax, obſervations on the 73

Incontinence of urine, caſe of 423

Incontraction of an artery, on the 2

Indian army, luxury of an 404

Indians, their uſe of pot-aſh inſtead of ſalt 475

Inflammation, ſtrangulation produced by 248

Influenza, Dr. Beddoes's collection of papers on 73, 97, 193, 289, 385, 517

———, Dr. Nott on the 80

———, Mr. Rodman on the 46

———, Mr. Luſcombe on 127

———, Mr. Dunning on 129

———, Mr. Peaal on 231

———, Mr. Hunter on 233

———, Mr. Cuming on 312

———, Mr. Stables on 355

———, Mr. Weber on 516

Inoculation hoſpitals, report of the committee of 191

———, report on the cow-pock 187

——— of ſheep with cow-pock 192

———, Mr. Dennet on 321

———, on the goat-pock 339

———, on vacciolous 474

Intermittent fever, caſe of a variety of 427 541

———, on the uſe of gelatine in 323

Inteſtinal vermes, a remarkable ſpecies of 547

Inſtrument, deſcription of a new tooth 241

——— for facilitating evaporation, account of an 479

Inſtruments for removing the cataract, deſcription of *(with a plate)* 65

Iron, on the volatile ſtate of 185

Irritability and excitability, on the cauſes of 25

Jamaica, progreſs of vaccine inoculation in 544

Jardine's, Mr. deſcription of a new tooth inſtrument 241

Jenner, Dr. communications to 93

Johnſon's, Mr. Hiſtory of Animal Chemiſtry, announced 480

Johnſtone's, Dr. Account of the Diſcovery of the Power of Mineral Acid Vapours to deſtroy Contagion, reviewed 78

Joints, on caries of the 5[illegible]7

Jones, Mr. on the influenza 107, 113

Jullion, Mr. on hydrocephalus internus 150

Kilkenny, epidemic at 297

King's, Mr. caſe of ſpina bifida, *(with a plate)* 237

Kinglake, Dr. on the influenza 306

———, on reduced temperature 3[illegible]7

Kinglake, Dr. his correction of a misstatement 530

Kirby-Moor-Side, epidemic at 390

La Grave's experiments with the oxydated disks of the Galvanic pile 63

Lawrence, Mr. on the influenza 199

Laxatives, their use in puerperal fever 14

Lectures on the vaccine pock inoculation, plan of 190

——— announced. Mr. Cline and Mr. Cooper's on anatomy and the operations of surgery, 285. Drs. Babington and Curry on the practice of medicine, *ib.* Dr. Babington and Mr. Allen on chemistry, *ib.* Dr. Curry on the theory of medicine and materia medica, *ib.* Dr. Haighton on midwifery and physiology, *ib.* Mr. A Cooper on the principles and practice of surgery, *ib.* Drs. Roberts and Powell on the theory and practice of medicine, 286. Dr. Roberts's clinical, *ib.* Mr. Abernethy on anatomy and physiology, *ib.* Mr. Macartney on ditto, *ib.* Dr. Powell on chemistry and the materia medica, *ib.* Dr. Thynne on midwifery, &c. *ib.*—Mr. Headington and Mr. Frampton on anatomy and physiology, *ib.* Dr. Pearson on therapeutics, practice of physic, and chemistry, *ib.* Dr. Bradley on the theory and practice of medicine, *ib.*—Dr. Batty on midwifery, *ib.* Drs. Dennison & Squire on ditto, *ib.* Dr. Clarke on ditto, *ib.* Mr. Blair on physiology, 287. Mr. Wilson and Mr. Thomas on anatomy, &c *ib.* Mr Taunton on ditto, *ib.* Mr. Chevalier on surgery, *ib.* Mr. Carpue on anatomy and surgery, *ib.* Mr. Lynn and Mr. Carlisle on surgery, 384 Dr. Badham on chemistry, *ib.* Mr. Pearson on surgery, *ib.* Mr. Thomas on ditto, *ib.* Mr. Macartney on comparative anatomy, *ib.* Mr. Moore on the structure and diseases of the teeth, *ib.* Mr. Fox on ditto, 480.

Lee, Mr. on the influenza 99

Leeds, epidemic at, 194, 518

Leeson's, Mr. case of semiosseous tumour 162

Leg, case of ulcerated 8

Library, on the medical 334

Lichen islandicus, benefits of in asthma 379

Limbs, on the injuries of the lower 88

Littlehales, Dr. on the influenza 386

Liver, cases of abscess of the 1, 489

Lochia, on a stoppage of the 13

Lomas, Mr. on the influenza 198

London, account of diseases in an eastern district of 7[f], 180, 276, 378, 456, 566

Lozenges, on tolu, *(note)* 352

Low, Mr. on metallic casts of the ear, 357

Ludlow, epidemic at 219

Lungs, on stony concretions coughed up from the 327

Luscombe, Mr. on the influenza 127

Lute for chemical purposes, account of a 192

Luxation of the femur, case of the 494

Lynn, epidemic at 405

Lynn's, Mr. lectures announced 384

Macartney's, Mr. lectures announced 286, 384

Madras, progress of vaccine inoculation at 93

Magennis, Dr. on the influenza 122

Man, epidemic in the Isle of 529

Manchester, epidemic at 211

Mania, case of 50

Marcet, Dr. letter from Dr. De Carro to 242

March, state of the weather in 146

Marcus's, Dr. Galvanic experiments 333

Marshall, Dr. on the influenza 405

Marson, Mr. on securing the elastic catheter 174

———, on the furniture of a sick room 175

———, on church-yard infection 177

———, on the delivery of the placenta 433

Marten's, Dr. on the medical application of Galvanism 330

May, Dr. on the influenza 291

Mead, Dr. original letters of 48

Measles, on the 487

Medical directions for the use of navigators and settlers in hot climates 83

——— ethics, account of Dr. Percival's 96

——— library, remarks on the 334

——— lectures, see Lectures

——— and Physical Intelligence 93, 190, 283, 379, 474, 574

Medicine act, observations on the amended 165, 258, 347

Melhere, M. De La, on irritability and excitability 25, 252

Melhuish, Mr. on the influenza 304

Mercury, effects of oxygenated muriat of 283

Metal, account of a new 96

Metallic casts of the ear, observations on 171, 357, 359

——— stimulus, observations on 54

——— cobalt, method of obtaining 95

Metford, Dr. on the influenza 108

Millet, Mr. on cold water in the gout 254

Mineral acid vapours, on their power to destroy contagion 78

Monstrosity, case of 172

Moodie's, Dr. case of abscess in the breast 414

Moor, Mr. on the vascularity of the teeth 360

———'s lectures announced 384

Moreau, Dr. on caries of the joints 557

Morrison, Dr. on the influenza 222

Moffman, Dr. on the nature and cure of the puerperal fever 11
———, on the influenza 387
Motion, on the caufe of animal 253
——— of the blood in the arteries, and of water in troughs and conduits, analogy between 284
Muriatic æther, on preparing 94
Mufcles of animals, on the influence which produces contractions in the 53
Mufcles, on the effects of eating 482

Natural Hiftory of the Human Teeth, reviewed 77
Navigators, medical directions for 83
Nettle-rafh fever, obfervations on the 481
Newry, epidemic at 295
Norfolk, improved ftate of the county of 405
———, (North America) hiftory of the yellow fever at 266
Nott, Dr. on the Influenza, reviewed 80

O'Berne on the fevers of hot climates 36
Obfervations on Diarrhœa and Dyfentery, as they appeared in the Britifh Army during the Campaign in 1781, reviewed 279
Œfophagus, apparatus for removing extraneous bodies from the 479
Omentum, cafe of a wound in the 356
———, cafe of fuppuration in the 29
Opifthotonos, cafe of 492
Opium, on its ufe in puerperal fever 16
———, experiments relative to the analyfis of 42
———, on its effects on the human body 435, 501
———, remarks on the modus operandi of 532
Offeous cavities of the ear, on metallic cafts of 171
Ottery St. Mary, influenza at 293
Oxyds, on thofe of the metallic difks of the Galvanic pile 63
Oxygenated muriat of mercury, effects of 283
Oxygen, on its efficacy in fyphilis 90

Palladium, a new metal, defcribed 96
Paralyfis, the rhus radicans recommended in 480
Paralytic difeafes, efficacy of Galvanifm in 60
Patterfon's, Dr. cafe of angina pectoris 64
Paul, Mr. on artificial Cheltenham water 574
Paulus on furgical operations 6
Payffe's, Mr. lute for chemical operations 192
Peal, Mr. on the influenza 233
———, on the cure of chancre 499
Peach-tree, Pruffic acid in the diftilled waters of the flowers of 95
Pearfe, Mr. on the influenza 203
Pearfon's, Dr. plan of lectures on the vaccine inoculation 100
——— medical lectures announced 286
——— Mr. furgical lectures announced 384
Pembroke, epidemic at 113
Percival's, Dr. medical ethics, account of 96
Petechiæ fine febre, cafes of 444
Pew, Mr. on the influenza 523
Phthifis pulmonalis, fuccefsfully treated by digitalis 551
Phyfiology, review of Richerand's Elements of 567
Pile, on the Galvanic 63
Placenta, on the delivery of the 433
Plan of lectures on vaccine inoculation 190
Plants, on diftilled waters of inodorous 95
Platt's, Mr. Inquiry into the Efficacy of Oxygen in the Cure of Syphilis, reviewed 90
Plymouth, on the epidemic at 122, 133, 291
Pneumonic inflammation, cafe of a 29
Pontier's, Mr. cafe of epilepfy 51
Portfmouth (North America) bill of mortality at 478
Pot-afh ufed with animal food 475
Powell's, Dr. lectures announced 286
Practical Obfervations on Surgery, reviewed 369, 466
——— the Management of Ruptures, reviewed 375
Pregnancy, on an incarcerated hernia during 4
Prieftley, Dr. on finery cinder and charcoal 273
Pruffic acid, difcovery of the 95
——— method of obtaining 479
Pterygium, defcription and cure of 563
Puerperal fever, on the nature and cure of 11
Pupilla claufa, mode of treating 565
Quaffia, its ufe in fevers 39
Queftion, Prize, of the Academy of Gottingen 383

Radix lopez recommended in colliquative diarrhœas 477
Ramfay's, Dr. Charlefton Medical Regifter reviewed 468
Reddlefden, Mr. on the influenza 213
Redfearn, Dr on the influenza 211
Reece, Mr. on the influenza 229
Reid's, Dr. cafe of trifmus traumatica 345
Remmett, Dr. on an epidemic 133
Renaud, M. on the effects of arfenic 575
Renoult's, M. cafe of hernia 248
Report on the cow-pock inoculation 137
——— of the fmall-pox and inoculation hofpitals 191

Rewell, Mr. on the influenza 409
Rheumatifm, on cold water in 256
Rhus radicans, benefit of the 480
Ricards's, Mr. report of furgical cafes at the Finfbury Difpenfary, 74, 178, 275, 376
Rice, Mr. on the influenza 112
Richerand's Elements of Phyfiology, reviewed 567
Richter's Method of obtaining tungften 479
Ring, Mr. on vaccinators 159
———, on inoculation 421
———, treatife on the cow-pox, part ii. reviewed 473
———, on the cow-pox 544
Ring-worms, cured by the rhus radicans 480
Roberts's, Dr. lectures announced 286
Robertfon's, Mr. cafe of fuppuration in the omentum 29
———, cafe of hernia congenita 33
Robinfon, Dr. on the influenza 109
———'s cafe of abfcefs of the liver 489
Rodman, Mr. on the influenza 46
Rolfe, Mr. on the influenza 98
Ruptures, ufeful hints to thofe afflicted with 92
———, practical obfervations on the management of 375
Rufh, Dr. on the cure of difeafes by the extraction of decayed teeth 431
Ryan, Dr. on the influenza 296, 305

Sacco, Dr. on vaccination 93
Sainsbury, Dr. on the influenza 115
Salius, on an epidemic 142
Sandford, Mr. on the influenza 223
Scabies, muriatic acid ufed in the cure of 576
Scarifying, on the ufes of 425
Scarlatina anginofa, prevalence of 76, 276
Scarlet fever, on the prevention of 457
Scarpa, Mr. on difeafes of the eyes 561
Schaub's, Dr. method of obtaining pruffic acid 479
Schrader's, Mr. difcovery of the pruffic acid 95
Scott, Dr. on the influenza 529
Selby, epidemic at 393
Selden's, Dr. hiftory of the yellow fever 266
Semiofteous tumour, cafe of a 163
Senfation in animals, of the caufe of 253
Shann, Mr. on the influenza 392
Sheep inoculated with the cow-pox 192
Sheffield, epidemic at 387
Sheldrake's, Mr. hints to thofe afflicted with ruptures, reviewed 92
Short, Mr. on the influenza 101
Shrewfbury, epidemic at 217
Sierra Leone, on the climate and difeafes of 85

Simmons's, Mr. obfervations on felect fubjects in furgery 1
———, on a cafe of abfcefs of the liver ib.
———, on an incontraction of an artery 2
———, on an incarcerated hernia during pregnancy 4
———, on a wound of the trachea 5
———, on a cafe of ulcerated leg 8
———, on a cafe of early cataract 10
Simpfon, Mr. on the influenza 101
Simpfon's, Mr. cafe of compound fracture 316
Skrimfhire's Chemical Effays announced 480
Small-pox hofpitals, report of 191
Smart, Mr. on the influenza 393
Smith, Mr. on the influenza 103
———, defcription of an improved trephine 239
Smyth's, Dr. formula of ufing muriatic acid vapour 79
Southall, Mr. on the influenza 102
Spalding's, Mr. bill of mortality at Portsmouth (North America) 478
Spaniard, account of an extraordinary 380
Speer, Dr. on the influenza 408
Spina bifida, cafe of (*with a plate*) 237
Spirits, diftilled, quantity of ufed in America 476
Squire's, Dr. lectures announced 286
Stables, Mr. on the influenza 355
———'s, Mr. cafe of a wound in the omentum 356
Stanley, Mr. on the influenza 527
Steel, Mr. on the influenza 228
Stony concretions coughed up from the lungs 327
Stringham, Dr. on the effects of oxygenated muriat of mercury 283
———, on inteftinal vermes 547
Sunderland, epidemic at 399
———, report of difeafes in the workhoufe at 454
Suppuration in the omentum, cafe of a 29
Surgery, obfervations on felect cafes of 1
———, remarks on Bell's 154
———, practical obfervations on, reviewed 369, 466
Surgical cafes in Finsbury Difpenfary, monthly report of 74, 178, 275, 376
Suroxygenated muriatic acid, ufed in fcabies 576
Swanfea, epidemic at 201
Symes, Mr. on the influenza 228
Symonds, Dr. on the influenza 224
Syphilis, on the efficacy of oxygen in 90

Tadcafter, epidemic at 392
Tangiers, defcription of 381
Taunton's, Mr. lectures announced 287
Taylor, Mr. on the influenza 225

Teeth, Natural Hiſtory of the Human, reviewed 77

——, deſcription of an inſtrument for the extraction of 240

——, on the vaſcularity of the 360

——, on the cure of diſeaſes by the extraction of decayed 431

Temperature, on the benefits of reduced 254, 357

Thackeray, Dr. on the uſe of cold water in convulſions 420

Thiebaut, Mr. on the blue diſeaſe 575

Thirſk, epidemic at 395

Thirſt, on the cauſe of 20

Thomas's, Mr. lectures announced 287, 384

Thorax, caſe of a wound penetrating the abdomen and the 250

Thoreſby, Mr. on the influenza 409

Thorp, Dr. on the Influenza 219

Thynne's, Dr. lectures announced 286

Tickell, Mr. on the influenza 105

Timbrell's, Mr. Obſervations on the Management of Ruptures, reviewed 375

Tinea, caſe of 542

——, benefit of muriatic acid in 576

Tolu lozenges, obſervations on *(note)* 352

Trachea, on a wound of the 5

Trephine, deſcription of an improved *(with a plate)* 239

Trichiaſis, deſcription and cure of 562

Triſmus traumatica, caſe of 345

Tromſdorff's method of obtaining pure metallic cobalt 35

Trotter, Dr. on drunkenneſs and its effects on the human body 382

Trye's, Mr. illuſtration of ſome of the injuries to which the lower limbs are expoſed 88

Tubingen, medical eſtabliſhments at 480

Tudor, Mr. on the influenza 226

Tumour, caſe of ſemi-oſſeous 162

Tungſten, method of obtaining 479

Turton, Dr. on the influenza 201

Ulcerated leg, caſe of 8

Ulcers of the cornea, deſcription and cure of 563

Urethra, on ſecuring the catheter in the 174

Urine, caſe of incontinence of 423

Urticaria, on the 482

Vaccination, Mr. Ring on 159

————, Dr. De Carro on 242

Vaccinator, on the 41

Vaccine virus, on preſerving 40

——— inoculation, progreſs of in India 93

——————, Dr. Sacco on 93

——————, plan of lectures on 190

——————, reports on 187, 191, 288

Vaccine inoculation in Barbadoes, account of 424

——————, preventive againſt the plague 474

Vagina, caſe of an extraneous ſubſtance lodged in the 491

Valli's, Dr. experiments on the plague 475

Van Den Boſch, Dr. on the radix lopez 477

Vaſcularity of the teeth, obſervations on the 360

Vaughan, Dr. on the autumnal fever in America in 1802 365

Vazie's, Mr. inſtrument for facilitating evaporation, account of 479

Vermes, on inteſtinal 550

Veſicæ loturæ, on the practice of, in the cure of diſeaſed bladders 82

Vitriol, on the uſe of white, in agues 496

Ward, Mr. on cancer 448
———— on the influenza 521
Warts, on the removal of 179
Waſſe, Mr. on the influenza 395
Water, analogy between the motion of the blood and that of 284
——— on cold in convulſions 410
Weather, ſtate of the 146
Weber, Mr. on the effects of opium on the human body 435, 501
———— on the influenza 516
Weſton, Mr. on the Cow-pock 544
Whately, Mr. on the influenza 520
Whitehaven, epidemic at 111
Whitehead, Dr. on the yellow fever 266
Whitely, Mr. on the influenza 393
Whitlock, Mr. on the influenza 293
Wichmann, Dr. on the nettle-raſh fever 481
Wilkinſon's, Mr. experiments on the broad leafed willow bark 374
———— on the influenza 399
———— report of diſeaſes in the workhouſe at Sunderland 454
Williams, Mr. on the influenza, 230, 289, 294
Wilmington, (N. Amer.) hiſtory of the yellow fever at 266
Wilſon, Dr. on digeſtion 318
——— Mr. on the medical library 334
———'s lectures announced 287
Winterbottom's, Dr. medical directions for navigators and ſettlers in hot climates 83
Wiſbich, epidemic at 204
Withers Wm. extraordinary narrative of 472
Wurtemburg, Duke of, eſtabliſhes an hoſpital at Tubingen 480

Yellow fever, deſcription of the 87

Yellow Fever, ſhort hiſtory of that which prevailed in Norfolk, North America 266

Yeo, Mr. on the influenza 224

Yonge, Mr. on the influenza 221

York, epidemic at 389

Younglove's, Mr. analogy between the motion of the blood and that of water in troughs 284

Zadig, Dr. on Galvaniſm 59

### REFERENCES TO THE PLATES.

Prof. Arnemann's Instruments for the Cataract — — — 65
On the Disease of one of the Bursæ Mucosæ — — — *ib.*
Mr. Giraud's Case of Monstrosity — — — — — 172
Mr. King's Case of Spina Bifida — — — — — — 237
Mr. Smith's Improved Trephine — — — — — 239
Mr. Jardine's new Tooth Instrument — — — — — 242

